# A qualitative exploration of post-acute stroke participants’ experiences of a multimodal intervention incorporating horseback riding

**DOI:** 10.1371/journal.pone.0203933

**Published:** 2018-09-20

**Authors:** Petra Pohl, Gunnel Carlsson, Lina Bunketorp Käll, Michael Nilsson, Christian Blomstrand

**Affiliations:** 1 Department of Clinical Neuroscience, Institute of Neuroscience and Physiology, Sahlgrenska Academy at University of Gothenburg, Gothenburg, Sweden; 2 Department of Activity and Health, and Department of Medical and Health Sciences, Linköping University, Linköping, Sweden; 3 Centre for Advanced Reconstruction of Extremities, Sahlgrenska University Hospital/Mölndal, Mölndal, Sweden; 4 Department of Health and Rehabilitation, Institute of Neuroscience and Physiology, Sahlgrenska Academy at University of Gothenburg, Gothenburg, Sweden; 5 Hunter Medical Research Institute, University of Newcastle, Newcastle, Australia; 6 Stroke Centre West, Sahlgrenska Academy at University of Gothenburg, Gothenburg, Sweden; University of Birmingham, UNITED KINGDOM

## Abstract

**Background:**

Multimodal rehabilitation interventions delivered in late phase of stroke recovery involve physical (motor and sensory), social, and cognitively challenging activities. Horseback riding can be incorporated within such interventions, leading to meaningful long-term improvements when applied to individuals with moderate levels of disability. There is a lack of research illuminating stroke survivors’ experiences and perceptions of horseback riding in the context of multimodal interventions.

**Aim:**

To explore stroke survivors’ experiences of participation in a multimodal group-based intervention that included horseback riding.

**Methods:**

An explorative interview study was conducted with individual face-to-face interviews performed on a single occasion, utilising a semi-structured interview guide. Eighteen participants were purposively selected from a larger trial (mean age 62, 12 men, 6 women) within four weeks after treatment completion. The interview duration was between 17 and 50 minutes. The data was analysed using a qualitative content analysis method.

**Findings:**

Four broad themes were identified from the analysis. These themes were: *transformative experiences*; *human–horse interaction*; *togetherness and belonging*; and *the all-in-one solution*. Interacting with the horse and peers had a profound emotional impact on the participants. The participants also reported having learned new skills, increased self-efficacy and self-esteem, and improvements in balance and gait, all of which could be transferred to everyday life. The horse itself played a central role, but other components, such as the other group members, the instructors, and the challenging tasks on the horseback, were also important.

**Conclusion:**

A multimodal rehabilitation intervention that includes horseback riding may provide stroke survivors in a late phase of recovery with rich pleasurable experiences that may have life-changing and profound impacts on their emotional and physical state.

## Introduction

Stroke rehabilitation aims to optimise stroke survivors’ life satisfaction and to support the possibility of leading an independent everyday life [1]. Rehabilitative actions are usually initiated in the acute phase after stroke and typically involve active mobilisation and task-oriented exercises delivered by a stroke team [2]. Although there is consensus that early rehabilitation is preferable to late onset therapy [3], there is a need for continuation of rehabilitation in the chronic phase [4, 5]. Engaging in programmes with concurrent physical (sensory and motor), cognitive, and social activity may boost experience-driven neuronal plasticity and restitution of function in stroke-damaged areas [3, 6, 7].

Increasing evidence suggests that rehabilitative activities in stimulating environments can provide functional improvements also in a late phase of recovery [8–10], but the vast majority of these studies are based on animal models. The real challenge is to translate these principles into clinical settings. Recently, two restorative group-based rehabilitation programs building on the principles of multimodal stimulation with multiple elements were evaluated in a three-armed randomised controlled trial with stroke survivors in a late phase of recovery. Within this trial, rhythm-and-music or horseback riding were integrated components, together with challenging tasks and opportunities to socialise with peers [11, 12]. The results confirmed that it is possible to reach long-lasting functional improvements even several years after the stroke onset by using multimodal interventions. Both groups improved significantly, but results were more favourable in the horseback riding intervention: after six months, 56% in the horseback riding programme experienced that their stroke recovery had further progressed, compared with 22% of participants in the control group [11]. Furthermore, objective measures confirmed functional improvements in balance, working memory, gait, and grip strength in both groups [11]. The study represents an important step towards understanding the potential of complex and multimodal interventions [11].

The decision to incorporate horseback riding in the multimodal intervention was based on its proposed therapeutic benefits. Various horse-based activities and therapies have been systematically studied in older community-dwelling people [13], multiple sclerosis [14, 15], mental disorders [16, 17], and in children with cerebral palsy [18, 19]. With respect to stroke, it was shown that 30 minutes of horseback riding once a week improved motor impairment of lower limbs, independence of ambulation, gait performance, and quality of life, when combined with conventional physiotherapy [20, 21]. Mechanical horses have also been shown to be beneficial for people with stroke, when the aim is to improve balance and asymmetric body weight bearing [22, 23]. An action research project in Sweden with 24 individuals (2 with stroke) showed that equine assisted therapy (EAT) did alleviate severe back pain [24]. It also identified EAT as a transitional process incorporating four dimensions, i.e. body awareness, competence, emotion, and environment. The authors discussed the importance of positive feelings and meaningful activities in the enriched environment that the horse and stable offer [24]. Close relationship with a horse, including a properly aligned position on the horse’s back, may also contribute to the development of non-verbal communication skills [24], suggesting that horse-based activities may be beneficial for people with aphasia.

Due to the complexity of multimodal stimulating interventions and the multitude of experiences that may be involved, it is recommended that the subjective in-depth perspectives of such interventions be explored, in addition to obtaining objective measurements [25, 26]. In this regard, a qualitative approach may be appropriate for generating complimentary person-centred knowledge not revealed by quantitative data [27–29]. Within the field of medicine, only few qualitative studies have been identified exploring the riders’ perspectives. Apart from the study concerning people with back pain [24], one qualitative study demonstrated psychosocial benefits for five people with psychiatric disabilities [17], and one study including 15 people with various disabilities (two with stroke) revealed that horseback riding may have a positive influence on the participants’ identity construction [30]. There is, however, a lack of research illuminating stroke survivors’ experiences and perceptions of horseback riding in the context of multimodal interventions. A qualitative study in this context may better capture experiences that reflect the complexity of the intervention design. The aim of the present study was thus to explore stroke survivors’ experiences concerning participation in a multimodal group-based horseback riding intervention.

## Methods

### Study design

This qualitative study consisted of semi-structured interviews with participants selected from a 12-week multimodal intervention to allow in-depth exploration of participants’ perspectives. The study followed recommended guidelines for qualitative inquiry within the field of medicine and public health [28, 31]. The reporting of the study has been done according to the COREQ criteria [32] (S1 Table).

### Theoretical framework

In order to obtain a deeper understanding of the dynamic interactive experiences of taking part in a multimodal rehabilitation programme incorporating horseback riding, the philosophical position underlying this study relied on an interpretivist orientation to qualitative inquiry. According to Guba & Lincoln (1985), the epistemological position of interpretivism is subjectivism, and the ontological position is relativism, where reality is seen as subjective and varying from person to person [33]. In addition, an interactionist perspective was adopted as methodological approach where people are seen as actively participating in the creation of their own development through interaction with the social world [34, 35].

### Study context

For this interview study, a sub-population of 18 participants was selected among 123 individuals consecutively recruited to participate in an intervention trial in a late phase of stroke recovery. The inclusion process for this trial has been thoroughly described in the study protocol [12]. After inclusion, the participants were randomised to either the horseback riding group, to a rhythm-and-music based group, or to a control group. The randomisation was stratified with respect to gender and hemispheric location of the stroke [12]. The contents of the horseback riding intervention are shown in the supplementary file ‘S2 Table’. In all, there were eight separate groups with between four and seven participants in each group. The rhythm-and-music intervention is described elsewhere [12].

All participants in the active interventions were asked if they would agree to be interviewed, with the motivation that physical assessments and questionnaires would not sufficiently capture their personal experiences and perceptions of taking part in the multimodal interventions. All participants in the horseback riding therapy group agreed to be contacted, except three. The reasons for refusal are outlined in the supplementary file ‘S1 Appendix’. Prior to the large trial, ethical approval was obtained from the Regional Ethical Review Board in Gothenburg (ref. no. 698–09). The study was conducted in accordance with relevant ethical guidelines including written informed consent from the participants [11]. Informed consent was obtained by experienced health professionals according to specified inclusion and exclusion criteria that assured capacity to consent. The study was registered on ClinicalTrials.gov, identification number NCT01372059.

### Participant selection

#### Sample size

As the research question was considered to be narrow in scope, it was estimated *a priori* that between 15 and 20 participants would be sufficient to capture a variety of experiences, instead of using data saturation to determine sample size. This is seen as a pragmatic approach to sample size [31].

#### Sampling and method of approach

A list of all participants within the horseback riding group containing basic demographics was distributed to GC, an occupational therapist with several years of experience from conducting qualitative research. Based on the need for a rich diversity in age, sex, stroke location and stroke severity, GC purposively selected participants to contact. Representatives from all eight groups were selected. Efforts were made to also include people with aphasia, despite their lack of ability to fluently express themselves verbally. Eighteen participants were identified who represented the required diversity and were contacted by telephone by GC within four weeks after treatment completion. All participants (twelve men; six women; mean age 60.3 years, range 51 to 74 years) agreed to be interviewed.

#### Setting

All interviews were conducted at a rehabilitation facility at Sahlgrenska University Hospital in Gothenburg, Sweden. This out-patient setting was chosen to ensure confidentiality in a relaxed atmosphere. During one of the interviews, a personal assistant, employed by the participant for verbal support, was also present. This interview was also visually recorded to later facilitate the verbatim transcription. Table 1 presents demographic details of participants.

**Table 1:** Summary of all interview participants showing demographics, level of disability, presence of aphasia, and perceived improvement, based on a dichotomisation of participants’ ratings on a 100 mm visual analogue scale, where 10 mm increase was considered to be a perceived improvement (yes).

Code	Sex	Age at inclu-sion	mRS*	Aphasia	Time since stroke onset/years	Perceived improvement
P1	Male	55	3	No	3.1	No
P2	Female	59	2	No	3.2	Yes
P3	Male	58	2	No	5.1	Yes
P4	Male	66	3	No	2.9	Yes
P5	Male	59	2	No	2.8	No
P6	Male	74	3	No	3.7	Yes
P7	Female	66	3	No	2.7	No
P8	Male	60	2	No	3.8	No
P9	Male	70	3	No	2.2	No
P10	Female	63	2	No	2.3	No
P11	Male	56	2	No	1.5	Yes
P12	Male	61	3	Yes	9.9	Yes
P13	Male	51	2	Yes	3.1	No
P14	Female	59	3	Yes	3.0	No
P15	Male	66	2	Yes	4.9	No
P16	Female	67	2	Yes	1.8	No
P17	Female	53	3	Yes	2.2	Yes
P18	Male	53	3	Yes	2.0	No

* modified Rankin Scale: An ordinal disability rating scale ranging from zero to 6 (0 = no symptoms; 6 = dead). mRS grade 2 = *slight disability*: unable to carry out all previous activities but able to look after own affairs without assistance; mRS grade 3 = m*oderate disability*: requiring some help, but able to walk without assistance

#### Data collection

Data was collected through individual face-to-face interviews on a single occasion between December, 2010 and August, 2013 using a semi-structured question guide (Table 2). All interviews were audio recorded. Sixteen interviews were conducted by GC and seven with the assistance of a speech therapist specialised in aphasia who facilitated interviews with supportive conversational techniques (e.g., pictures of the horses and the riding centre) [36]. Two interviews were conducted by the speech therapist alone. One pilot interview was carried out by GC, but no adjustments were considered necessary. The pilot study was thus included in the analysis. In all interviews, the initiating prompt was “Can you, in your own words, describe how you experienced taking part in the horseback riding therapy?”, followed by frequent promptings such as: “If so, in what way?” to encourage the participants to elaborate their thoughts. This approach suited our objective of obtaining in-depth data. No repeat interviews were conducted. No field notes were taken during the interviews. The duration of the interviews was between 17 and 50 minutes. Each interview was transcribed verbatim in Swedish by a person external to the research group and thus without influence over the research process. Transcripts were anonymised by a unique number prior to the analysis (P1 to P18). The transcripts were verified for accuracy by PP (a physiotherapist and trained medical secretary) by cross-checking the transcripts with audiotapes. No transcripts were returned to participants for comment or correction.

**Table 2:** Semi-structured question guide for the individual face-to-face interviews.

• Describe in your own words your experience of participating in the horseback riding group? Positive–negative experiences?
• Has this participation had any effect on your physical, psychological or social abilities?
• Has participation meant something for you in your contact with other people? In connection with horseback riding group? In other contexts?
• Has participation affected your activity performance in life in general?
• Has participation affected your life situation in general?
• Has participation affected your mood, quality of life, and beliefs about the future?

### Data analysis

The method used for data analysis was qualitative content analysis [37, 38], which is appropriate when the aim is to focus on the subjects as well as on the contextual meanings [37]. The emphasis lies on describing variations, e.g. similarities and differences within parts of the text [37, 39]. Qualitative content analysis may comprise both manifest contents (close to the text) and latent meanings (close to the lived experiences, but more distant from the text) [39]. In this study we have mainly described the manifest contents, which still also requires some degree of interpretation [39]. An inductive (data driven) approach was used in order to identify patterns of the content [39]. With an inductive approach, little or no predetermined theory, structure, or framework is used to analyse data [38]. This approach is recommended when there is little former knowledge about the phenomenon of interest [38]. Thus, the themes were derived from the data and not on the basis of previous knowledge or theories. The process of deriving themes was done using a step-wise procedure. The 18 transcribed interviews were considered as the unit of analysis. All interviews were initially read repeatedly and independently by PP and GC to start the process of identifying meaning units corresponding to the objective and to acquire a good grasp of the whole. Next, the same two authors compared their meaning units, and when differences appeared, they were discussed until consensus was reached. All text units (i.e., the interviews) were then transferred to a computer software system (Open Code 4.0, freely available from Umea University at http://www.phmed.umu.se/enheter/epidemiologi/forskning/open-code), and the meaning units were subsequently condensed into labelling codes by both authors together. A code is close to the text (manifest level) and describes the content of the meaning unit with a few words [37]. The codes were then grouped into themes with similar contents and labelled with a description of their contents. The themes, sub-themes, and examples of meaning units and codes are shown in the supplementary file ‘S3 Table’. The process of grouping codes into themes was conducted through several meetings between GC and PP. The final set of themes was presented along with the codes to the other authors (LBK, MN and CB) and determined by consensus in the research team during two study meetings.

### Trustworthiness and reflexivity

Qualitative research has been gaining acceptability in the medical literature [28]. In order for the reader to judge the scientific rigour of the findings, it is therefore important to establish trustworthiness [29]. As suggested by Lincoln & Guba (1985) [33], we have considered the following aspects of trustworthiness in relation to our study: credibility, transferability, dependability, and confirmability. To achieve credibility, efforts were made to include individuals with a wide variety of disabilities following the stroke, for example also including aphasia. This contributed to a richer variation of the phenomena under study. To further enhance credibility, quotations from the interviews are provided to illustrate and support the findings in order to highlight the relevance of the themes and categories. The use of direct quotes also increased the confirmability. To further enhance the trustworthiness, all quotes were translated by a professional English language editor.

To facilitate the transferability, we have tried to give a clear and distinct description of the context, selection, and characteristics of the participants, data collection, and process of analysis. Dependability relates to the ability of the researchers to be flexible and change perspective in accordance with the emerging process [29]. This was done by including a full audit trail of all steps and decisions taken during the process (S1 Appendix), a technique proposed by Lincoln & Guba [33].

Depending on positions and perspectives, different researchers might access different representations of the situation under study [40]. Members of the current research team represented different professions, allowing for triangulation of the analytical process. Methodological triangulation could have been further enhanced by including other data sources, for example by interviewing the instructors. Reflexivity is further discussed in the audit trail (S1 Appendix).

## Findings

**Fig 1:**
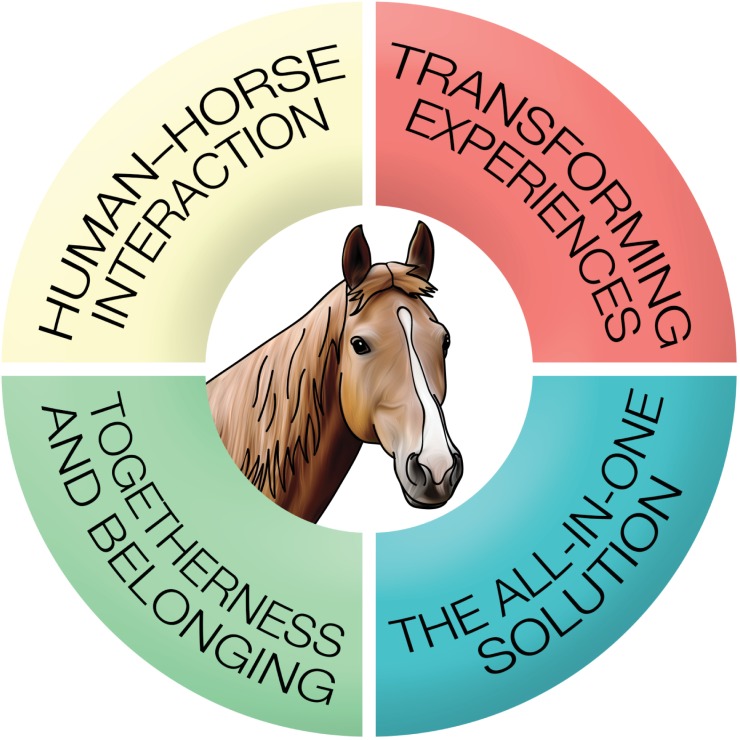
The four themes based on the findings, where the horse itself played a central part.

Eighteen participants aged 51 to 74 years took part in individual face-to-face interviews. The time elapsed since the stroke insult was on average 3.3 years, ranging from 19 months to 9.9 years. Participating in the multimodal intervention affected individuals in many dimensions, all of which revolved around the experiences with the horse (Fig 1). Analysis generated four broad themes: *transformative experience; human*–*horse interaction; togetherness and belonging;* and *the all-in-one solution*.

### Transformative experience

This theme described the deep experiences associated with the intervention and the changes that it brought about with regard to how participants viewed themselves and life after stroke, as well as actual functional improvements. All but seven of the participants had no previous experience with horses, and the experience of spending time with and riding the horse was well beyond their “wildest dreams” and very much beyond what they had expected: *I couldn’t have imagined in my wildest dreams that I would ever sit on a horse’s back*. *It was something that was completely beyond my imagination*. *[–––] So it was a fantastic experience*, *really*. [P9, male, 70 years]

Their preconceptions about the horse and horseback riding had changed profoundly from, for example, having seen the horse as a “hamburger” to a desire to own a horse farm: *To get a small farm and have a couple of horses*, *that would be something to wish for… If I had known this*, *I would have bought my uncle’s farm*. *But at that time*, *it was ‘never’ for me–a big no*, *no*! *Horses were hamburgers to me*! *Meat for McDonald’s*. *Now I even watch dressage*, *so I’m in a different world*. [P1, male, 55 years]

One participant described how he initially had felt a resistance to horseback riding and told about his experiences when he learned that he had been randomised to the horseback riding group: *I said to them*: *“You’re completely crazy” I said*. *“Are you going to get an old man of 70 up on a horse*?! *That’s really something*, *can’t you find some people who are a little bit younger instead*?*” They laughed at me then*. [P9, male, 70 years]

The participants initially felt that horseback riding was a dangerous activity, and that horses were big smelly animals that one should be wary of. At the same time, they were curious to try something new, and the appearance of a big horse was experienced with excitement, challenge, and respect. Mounting the horse resulted in an explosion of emotions. The participants were filled with positive feelings, gratitude, and humbleness. They felt that horseback riding did them good, that it was a warm and enlightening experience, and one of the best things that had ever happened to them. As one participant expressed it: *In my opinion this has been a positive experience*, *regardless of my state of health*. *I mean*, *seen from a slightly gloomier perspective*, *it was lucky that I got ill so I could take part in this [horseback riding]*, *if being ill was the excuse that was necessary*. *This has been great*, *so I have severe abstinence now that it’s all over*. [P5, male, 59 years]

The whole body was affected by this experience, and this was a particularly positive experience when one side was hemiplegic, for example: *I have never tried horseback riding before in my life*, *but once I got up on that horse and felt this… the whole body really got a kick*! *And I was able to lift my arm*, *I could get down off of the horse afterwards*, *I could do practically everything that I couldn’t do before*! [P6, male, 74 years]

The participants described noticeable physical and cognitive consequences of riding a horse, although it was also extremely tiring: *I was so devilishly tired in my head afterwards*! *Because it was like being in control of myself for just a few minutes… I was completely drained of energy and power after that*. *That’s why I felt that this was good training for me–this was exactly what my problem was*. [P13, male with aphasia, 51 years]

The most commonly perceived changes were improvements in balance and posture, which had led to noticeable impacts on everyday life activities, such as climbing stairs, being able to perform household activities independently, and no longer needing a walking aid. One participant with aphasia said: *I feel steadier now… You know*, *when I walk to the tram*, *I have to cross a street*, *and I couldn’t manage that before*. *I feel that I have become stronger with… well*, *that I have… [Interviewer*: *is it the balance*?*] Yes*! *Very much*! *[immediately]* [P16, female with aphasia, 67 years]

Another participant put it like this: *After six or seven times*, *I suddenly noticed that I was able to stand up in the bathtub without holding onto anything*! *Oh*! *I was so astonished when I stood in the shower*, *I thought to myself*: *“Oh my God*, *I’m not even holding onto something*!*” I had to call out to my husband and tell him*. *Just a small foolish thing for most people*, *but for me it was a huge thing that I could stand up all on my own*! [P7, female, 66 years]

The same participant also experienced memory related improvements: *And my memory was improved by the frequent mental repetitions that I did*, *when I was preparing myself for the next lesson*: *“Today I will trot*, *and how is this done again*?*” So*, *I had to think about it a bit in advance*.

Grooming the horse and holding the reins were also perceived as improving fine motor skills. This led to an enhanced motivation to continue with the horseback riding intervention.

Moreover, being able to manage the huge challenge of horseback riding also led to gradually increasing self-esteem and self-pride. Individuals who were initially afraid of performing the exercises on horseback, and in some cases felt challenged by trotting, experienced a growing sense of capability and success throughout the intervention, for example: *It’s very much about confirming that “I can*!*”*. *Obviously this has been a very good experience*! *[–––] It’s really a little extreme*, *when one thinks about it*: *sitting on a horse and holding onto it and trotting*, *and stuff*. *It gave a kick*, *and that’s probably important to a lot of people*. *[–––] It gave me an extra reinforcement of my self-esteem*. [P5, male, 59 years]

Observing the success of their peers with more severe stroke-related disorders also gave an insight:

*Above all*, *my self-esteem improved*, *that is the big thing*! *I felt that “I can do this*, *this is going well*!*” I was lucky enough to be one of the fittest… I felt that if the others in the group can manage*, *then I can too*! *And that*, *of course*, *gives you self-confidence*. [P8, male, 60 years]

This increased the overall courage to undertake bigger challenges in everyday life and even try new activities. One participant said: *I can do more than I thought*! *[–––] When I noticed that it went beyond my expectations*, *I realised that there might be other things that I could do that I didn’t think I could do*. [P7, female, 66 years]

### Human–horse interaction

This theme describes the physical and emotional closeness to the horse and the significant impact that the strength of the bond and interaction with the horse had on the participants. A sense of emotional bonding was formed between the participants and the horse during their time together, and the participants felt that a mutual understanding between the horse and the rider evolved. One of the participants even spoke of “falling in love” with this sensitive and beautiful animal: *I… fell in love… I think so… (trying to find the words) … no*, *oh*, *no [–––]*. *Well*, *it was very educational that a horse could mean so much to me*, *I didn’t think so*! *It’s their eyes*. *What eyes they have*! *[P16*, *female with aphasia*, *67 years]*

Touching the soft coat and inhaling the smell of the horse reinforced the sensation. Participants felt a strong relationship with the horse and were astonished that such an animal could mean so much and be experienced as a good friend. This new friendship stirred them very deeply and evoked strong emotions. One participant said: *I have never been so close to a horse before*, *it felt really great*. *It was almost as if the horse was*, *well not a person perhaps*, *but I wanted to hug the horse (laughs)*. *It felt like communing with an individual*! *[–––] The riding itself was also fun*, *but the most rewarding aspect was the horse*! [P8, male, 60 years]

Another participant described that the time in the stable was just as important as the riding itself:

*In order to ride*, *you can’t just sit on top of a horse*, *you also have to try to get close to the horse and get acquainted with it*. *When you groom it*, *you get this close contact and you get this feeling “it’s you and me working together”*. *[–––] That contact is just as important as getting up on the horse*. [P7, female, 66 years]

In addition, sitting only on a thin saddle pad gave an intense sensation and strong feedback from the horse´s moving body. The own body, that in many ways felt different after the stroke, was now perceived in new ways through the movements of the horse. The tight body-to-body connection was restful, and it became natural to follow the horse´s rhythmic gait, mimicking movements during riding, forcing the person to indulge in and completely rely on the horse, for example: *When you get up there*, *you must first of all learn about your body as a human being*, *how it works*. *Start learning how your body works*. [P1, male, 55 years]

*You learned how to balance*. *The horse moved and you had to continuously follow the horse’s movements*, *and that was really useful*. *You had to follow the rhythm of the horse*. *You couldn’t just sit there*, *you had to follow the horse’s gait [–––] That’s the whole point of riding bare-back*, *to gain the balance*. [P9, male, 70 years]

A feeling of mastery also developed as the ability to control the horse improved. The participants told of initially clinging to the horse, while at the end being able to manage the horse with ease and joy: *The first times were a bit hard*. *I had no real control*, *I thought*, *when I was sitting there on the horse… happy as long as I didn’t fall off*. [P13, male with aphasia, 51 years]

*I was helped to get up on the horse*, *I needed help to get the left leg over the withers*. *But then you could steer the horse by yourself*, *and that was really fun*! [P3, male, 58 years]

Learning was enhanced by observing the others, by listening to the instructor, and by trying themselves. The unpredictability of the horse was an additional challenge, as it could sometimes startle when exposed to a sudden noise or movement. This was experienced as pushing the limits and even led to a couple of fall accidents, but without injuries.

The horse itself was seen as a tutor, a friend, and a co-worker. Working together in a partnership with the horse was deeply rewarding. To be able to succeed with horseback riding, it was essential to have an interaction and collaboration between the horse and the rider. As one participant [P4, male, 55 years] described this: *It’s me and the horse who are doing this together*. *That felt really nice*.

The close contact with the horse also reinforced communication skills, in that the participants needed to be able to make the horse understand the participants’ intentions: *In the beginning I was just clinging to the horse to make sure I didn’t fall off*. *But once I found my balance and was comfortable sitting on horseback*, *I could do the other stuff too*. *[–––] I could make the horse understand my thoughts*, *and it did what I wanted it to*! [P5, male, 59 years]

One participant described how she needed to completely trust the horse in order to work together, and this involved giving away the authority and right to decide to the horse: *I’m so used to make my own decisions*, *but when I sat on Tasmina*, *it wasn’t up to me anymore*. *I had to give away the right to decide*, *and let her control how my body should work*, *because I had to follow her and not the other way around*. [P7, female, 66 years]

### Togetherness and belonging

A strong relationship between the group members developed during the intervention due to the amount of time spent together and the experience they shared. The opportunity to meet and interact with other stroke survivors was fruitful, both during the stable activities and the shared lunch. Even the time spent on the bench while waiting for one’s turn and observing the other group members ride was appreciated by most, although a few participants expressed that this wait was quite long and occasionally boring.

Several participants described how wonderful it was to follow the sometimes surprising progress of the others. This was seen as very inspiring. One woman told about a person with severe hemiplegia and aphasia who suddenly became more confident on horseback: *X [mentions a person’s name] who was in my group*, *he was terrified of horses*! *He had no previous riding experience*, *he was so frightened*, *and he was so good*. *That day when his fear let go*, *that was a fantastic sight*! [P7, female, 66 years].

The participants were also generous with positive affirmations and telling each other about the progress they had observed, for example: *We continuously told her that we noticed the improvements*, *and I think that spurred her to take on more challenges and not dwell upon “No*, *I can’t”*. [P7, female 66].

To meet other persons with the same condition also gave opportunities to discuss common issues and gave a feeling of being able to be oneself: *The social part was really important*! *You met some fellow sufferers*, *and we had a really good time*! *I think all of us enjoyed it*. *And eating lunch together afterwards was also very rewarding*. [P11, male, 56 years]

The rich opportunity to meet afterwards during lunch gave them time to reflect upon and process their experiences. It also gave a sense of landing after the intense activity. During lunch, when the staff and personal assistants were present, the participants were able to help each other analyse the riding experience and to provide generous feedback to each and affirmation of progress. The presence of the staff was highly valued in this process. *We tried to help each other a bit and talked about why things had happened*, *and why it didn’t go as planned and so on*. [P15, male with aphasia, 66 years]

Another participant said: *It was so nice and pleasant*, *because you also had a wonderful feeling of being able to land after the intense session*. *You were not in any hurry to go home*, *which also was valuable from a therapy point of view*. *We were encouraged to take it easy*. *This combination of working with the animals and then having time to deal with the experience afterwards was probably really good*. [P5, male, 59 years]

For those who normally felt isolated, it also provided good practice in socialising and developing interpersonal skills: *And then we had lunch*, *and we talked about what had happened*, *but also about other things*. *Because that’s what happens when you get to know each other better*, *you start talking about things in general*. *So*, *it also became good practice to socialise and to spend the time in a useful way*. *That was really good in my opinion*. [P15, male with aphasia, 66 years]

The participants with aphasia could practice speaking with people who had an understanding of their speech difficulties, which made them feel secure: *I think that everybody… when I talk… everyone is so positive*! *I need to take it slowly… [–––] And they just say*: *“take it easy*, *slow down*, *I have a mother or a father or”*, *so they always… (meaning that the others show an understanding)* [P17, female with aphasia, 53 years]

Another participant said: *We very rarely spoke about diseases*, *I don’t think that anyone was interested in this*. *We talked about anything else but diseases*. *[–––] We are often misunderstood*, *because we don’t have broken bones*. *It’s on the inside*, *and you sometimes avoid having difficult discussions because you know that you won’t find the words*. [P9, male, 70 years]

It was felt that the group dynamics was just as important as the horseback riding itself: *I think that the social bit worked really well*. *I believe that it’s important that the social part in the little group also works well*, *it’s not only the riding itself*! *The part when we ate lunch together was just as important as the riding itself*, *that’s what I think anyway*. *If that part doesn’t work*, *then the riding won’t work either*! [P9, male, 70 years]

One participant told that she had continued with horseback riding after the intervention, but had missed the social part very much and did not enjoy the riding as much.

### The all-in-one solution

This theme was a reflection upon the organisation of the intervention programme itself in relation to other training programmes, such as physiotherapy, as well as other aspects regarding the programme’s environment.

The arrangement of the programme that included stable activities, riding lessons led by skilled and devoted instructors, and lunch or refreshments with peers and staff was much appreciated by the participants. The social interplay and the practical activities were considered as equally important:

*Well*, *the all-in-one solution was great fun when we had lunch together and drank coffee and so on* … [P3, male, 58 years]

Some participants felt that it might be valuable to start with such a programme earlier in the rehabilitation process, but others confirmed that it was actually possible to make progress many years after stroke. It was stressed that others with disabilities, not only stroke induced, should be given the opportunity to participate in such a rich programme.

The instructors were present throughout the entire session. All participants spoke highly of the instructors. They were seen as devoted, professional, trustworthy, pedagogical, and competent, with great knowledge about both stroke symptoms and horseback riding. They gave the participants a feeling of equality–a feeling of being treated as adults. One participant said: *I think it was good that it wasn’t–excuse the expression–childish*! *We are in fact grown-ups*! *I think they managed to keep that distance*, *I really appreciate that*. *Instead we were able to meet like adults*, *and not looked down upon or treated as tiny little kids…* [P11, male, 56 years]

Another participant expressed that the instructors knew exactly what they were doing: *It was obvious that they knew what sort of people they were dealing with*, *they were very familiar with the problems one might have*. *That it can be so different from person to person*. *And they were also horse lovers*, *so they knew about those things too*. *They were able to “kill two birds with one stone”*, *so it was really good*. [P13, male with aphasia, 51 years]

It was experienced that the instructors tailored the exercises individually with a clear aim of progress, always challenging the limits of the ability of the participants. The participants were continuously given challenging tasks to perform, such as memorising a certain pathway, and this was especially appreciated. As one participant put it: *They increased the challenges little by little I would say*, *gradually we were expected to ride by ourselves*. *[–––] They also gave us different riding paths*, *or we could invent our own paths*, *and we had to memorise them and tell them how we planned to go*, *and then we had to actually do it*. *That was fun*, *and always a challenge*. [P3, male, 58 years]

Another participant put it like this: *And then of course we got better at it*, *so they raised the bar gradually*, *both with things that were difficult and not so difficult*. *All in all*, *it was terrific*! [P13, male with aphasia, 51 years]

Some of the participants expressed that these tasks were experienced as unvaried and occasionally boring, for example: *We were often asked to follow a certain pathway*, *and some of these became a bit too familiar*. [P15, male with aphasia, 66 years]

The instructors were also extraordinarily good at picking the right therapy horse for each participant’s specific needs. One participant who had observed the instructors test riding a couple of horses said: *Carefully selected*, *because of safety reasons*. *[–––] They had to try different horses to see if they would work or not*. *To see if they were eligible as therapy horses*, *they were not just any horse*. [P13, male with aphasia, 51 years]

The horseback riding itself was seen as a more challenging form of training, i.e. more complex and tougher, than ordinary rehabilitation activities. One participant said: *Lying on back chairs and doing that sort of exercise*, *I have done that privately*. *Doesn’t give me anything*! *And bicycling–completely useless*! *I used to be a professional bicyclist*, *you know*, *been cycling about 200 km a week or more*. *And I used to run 30 km a week*. *That sort of thing doesn’t do anything for me–but the horse*, *so damn good*! *I felt it all the way up to my neck*. [P6, male, 74 years]

One participant felt that both rehabilitation methods were equally important: *The horseback riding isn’t directed towards any particular body part … I mean like working out with a shoulder or an arm–I think that has to be done as well*. *In my opinion*, *that is very important*. *But with this training you have to handle the whole body*, *so it’s something completely different*. *But the one thing doesn’t rule out the other*! [P5, male, 59 years]

In comparison to home-based self-training, it was considered much better to train away from home twice a week, as home-based training was believed to be less effective. The same participant had this reflection: *Exercising on your own becomes very fragmented and difficult*. *But with horseback riding therapy you do it*! *You don’t have to think*, *you are in a different place with other people who do the same thing*, *and you just focus on the training itself*. *If you work out at home*, *it gets fragmented*. *Of course*, *you can do it*, *but the quality will be poor*. *[–––] That’s why it’s so important with organised training*.

## Discussion

This qualitative study showed that a group-based multimodal intervention that includes horseback riding and social activities may provide stroke survivors in late phase of recovery with rich pleasurable experiences having profound emotional and physical impacts. Participating in the intervention provided the participants with a plethora of intense sensual stimuli; touch, proprioception, vision, hearing, smell, and the vestibular system were all involved. The intimate closeness to the horse had a profound impact on the participants by affording a multitude of new sensations, such as those arising from stroking, grooming, and sitting on the large animal. The physical closeness helped to establish an emotional attachment to the therapeutic animal. Moreover, the close physical contact, in addition to the friendly and warm social environment within the horseback riding intervention, is essential for the release of oxytocin, a hormone that is associated with the “calm and connection” system [41]. This is something that is difficult to accomplish with a mechanical horse, although simulators have been found to be beneficial for stroke patients in other ways [22, 23].

Horses also stimulate communication skills, as the relationship between man and horse builds upon other values and demands than interpersonal connections [24]. Horses understand and respond to communication primarily through body language, so the participants must learn to become more aware of their bodies, their body language, and expression of emotion through their bodies [42]. The intervention also provided the opportunity to experience authentic relationships with peers and staff members, adding to emotional awareness, communication skills, and a sense of group cohesion. Experiencing the intervention in a social context with a small group of peers provided numerous opportunities for the participants to relearn essential skills for living and to employ activities such as observation, reflectivity, problem solving, and socialising.

Apart from emotional boosts, the participants also described improved body functions. Horseback riding is generally thought to affect many body systems, achieving improvements in endurance, flexibility, symmetry, and body awareness, and development of trunk and postural control. The mechanism through which the action of riding confers an effect is thought to be the equine walk, which provides a multi-directional input resulting in movement responses that closely mimic the movement of the pelvis during the normal human gait, a movement that is both rhythmic and repetitive [43]. The movements are continuously repeated in that the horse usually takes many steps during a session. Such repetition is considered to be an important component for driving neuroplastic changes in the brain [44]. In order to drive activity-dependent neuroplasticity further, it is also crucial that the activity is perceived by the individual as meaningful and significant. If the training is experienced as rewarding and stimulating in a positive way, it should increase the likelihood of neural changes [44]. Experiences of success may also help to generate motivation for further improvements and add to a feeling of hope [45].

Furthermore, the participants learned the difficult task to master a horse. This complex skill was, for most participants, completely new. The learning experience, as described by the participants, is in line with social cognitive theory [46]. According to this theory, people do not learn new skills solely by trying them and either succeeding or failing, but also by observing others. Learning is also affected by positive reinforcement. Through the horseback riding intervention, the participants experienced qualities such as self-pride, courage, and self-efficacy, which ultimately had an impact on their ability to constructively cope with new situations and everyday life problems. Self-efficacy, defined as an individual’s belief in his or her capability to organise and execute the courses of action that are required to achieve his or her desired outcomes, is an important aspect of emotional well-being [47], and consequently, building self-efficacy is often a goal of therapeutic interventions. According to Bandura (1997), self-efficacy beliefs influence an individual’s actions, effort, and resilience when faced with obstacles and failure, as well as the amount of stress and depression experienced during times of difficulty [47]. Outcomes of self-efficacy have been found to be very important for people with long-term neurological conditions; therefore, activities facilitating self-efficacy are valuable additions to community rehabilitation [48]. The value of horseback riding in building self-efficacy is well recognised and has been studied in various settings [17, 49, 50]. Learning how to relate to a horse and master its movements may very likely have the same effect on people in a late phase of stroke recovery.

The findings of this study are important for informing future rehabilitation development and delivery. A paradigm shift has occurred within stroke rehabilitation, and it is now believed that combining activities with information from different sensory modalities facilitates post-stroke recovery process in the late phase [4, 7]. The real challenge after stroke, however, is the lack of opportunity to participate in such activities. It is important to point out that a stroke need not affect a person’s response to pleasurable physical and social activities. It may therefore not be surprising that the intervention experiences had such a profound effect on the participants. It should be noted that the participants did not solely consist of persons who knew or believed that they would enjoy horseback riding, and the preconceived notions about horses were quite diverse among the participants. In addition, participants were randomised to either horseback riding or rhythm-and-music based intervention and were thus not able to choose which activity they would take part in.

The study has some limitations. The interviews were conducted over a long time span. This was due to the fact that it was difficult to recruit participants to the randomised controlled trial and because there was a pre-determined maximum number of persons who could participate in each horseback riding group. We believe that this prolonged process did not have a major negative impact on our findings. The small sample size limits the transferability, and therefore the findings are not transferable to the broader population of stroke survivors. In addition, we included stroke survivors with slight or moderate disability (modified Rankin Scale 2 and 3), and therefore, the findings are not generalisable to stroke survivors without apparent disabilities or those with severe disabilities. Inclusion of severely disabled survivors would, however, have increased the risk of adverse events and dropouts. Another limitation was that the inclusion of willing participants resulted in a 2–1 ratio of men to women, which may have resulted in a gender-biased view. However, a male overweight is seen for stroke in a middle-aged population. A strength of the study was the addition of a full audit trail, showing how the results were discussed until consensus between all researchers was reached, further enhancing the dependability. This audit trail also increased the confirmability, i.e., showing that efforts were made to keep our position as neutral as possible during the analytical process.

### Conclusion and relevance for practice

This qualitative study showed that rehabilitative activities based on principles of multimodal sensory stimulation that includes horseback riding may provide stroke survivors in late phase after stroke with rich pleasurable experiences that may have life-changing and profound emotional and physical impacts. This work adds to the body of knowledge about how such a program is perceived by stroke survivors and how multimodal therapies can be used in the community to facilitate the recovery process and build self-efficacy after stroke. Further research is warranted in order to explore long-term experiences, as well as assessing cost-effectiveness for providing this complex intervention in a late phase after stroke. The findings should also be confirmed in other cultural contexts and within other health care systems.

## Supporting information

S1 Table Reporting according to the COREQ criteria. (DOCX)

S2 Table Information about the horseback riding intervention. (DOCX)

S3 Table Summary of themes with examples of meaning units, codes, and sub-themes. (DOCX)

S1 Appendix Audit trail. (DOCX)
