# Supplementary material for: A qualitative exploration of post-acute stroke participants’ experiences of a multimodal intervention incorporating horseback riding
Source: PLoS One. 2018 Sep 20;13(9):e0203933. doi: 10.1371/journal.pone.0203933 (PMC6147507; doi:10.1371/journal.pone.0203933)
Supplement: S1 Table — (DOCX) [file pone.0203933.s001.docx]

| No. Item | Guide questions/description | Comment |
| --- | --- | --- |
| **Domain 1: Research team and reflexivity** |  |  |
| *Personal characteristics* |  |  |
| 1. Interviewer/facilitator | Which author/s conducted the inter­view? | GC with support of a speech therapist |
| 2. Credentials | What were the researcher’s creden­tials? *e.g., PhD, MD* | 2 MD and univer­sity profes­sors; all are PhD |
| 3. Occupation | What was their occupation at the time of the study? | Research Fellows |
| 4. Gender | Was the researcher male or female? | 3 female/2 male |
| 5. Experience and training | What experience or training did the researchers have? | > 20 years’ expe­rience from quali­tative research |
| *Relationship with participants* |  |  |
| 6. Relationship established | Was a relationship established  prior to study commencement? | None |
| 7. Participant knowledge of the interviewer | What did the participants know about the researcher? *e.g., personal goals, reasons for doing the research* | Broad outlines given in audit trail |
| 8. Interviewer characteristics | What characteristics were reported about the interviewer/facilitator? *E.g.,* *bias,* *assumptions, reasons and interests in the research topic* | Outlined in audit trail |
| **Domain 2: study design** |  |  |
| *Theoretical framework* |  |  |
| 9. Methodological orientation and Theory | What methodological orientation was stated to underpin the study? *e.g. grounded theory, discourse* *analysis, ethnography,* *phenomeno­logy, content analysis* | Interpretive interactionism; and aualitative content analysis |
| *Participant selection* |  |  |
| 10. Sampling | How were participants selected? *e.g. purposive, convenience,* *consecutive, snowball* | Purposive |
| 11. Method of approach | How were participants approached? e*.g. face‐to‐face,* *telephone, mail, email* | By telephone |
| 12. Sample size | How many participants were in the study? | Eighteen |
| 13. Non-participation | How many people refused to participate or dropped out? Reasons? | NA (No refusals nor drop-outs) |
| *Setting* |  |  |
| 14. Setting of data collection | Where was the data collected? *e.g. home, clinic, workplace* | At a rehabilitation facility |
| 15. Presence of non-participants | Was anyone else present besides the participants and researchers? | Yes, one personal assistant and one speech therapist |
| 16. Description of sample | What are the important characteristics of the sample? *e.g.* *demographic data, date* | People with stroke in a late phase of stroke recovery |
| *Data collection* |  |  |
| 17. Interview guide | Were questions, prompts, guides provided by the authors? Was it pilot tested? | Semi-structured interview. One pilot interview was conducted. |
| 18. Repeat interviews | Were repeat interviews carried out? | No |
| 19. Audio/visual recording | Did the researchers use audio or visual recording to collect the data? | Audio and one visu­al recording |
| 20. Field notes | Were field notes made during and/or after the interview or focus group? | No |
| 21. Duration | What was the duration of the  interviews or focus group? | Between 17 and 50 minutes |
| 22 Data saturation | Was data saturation discussed? | Yes |
| 23. Transcripts returned | Were transcripts returned to participants for comment and/or correction? | No |
| **Domain 3: analysis and findings** |  |  |
| *Data analysis* |  |  |
| 24. Number of data coders | How many data coders coded the data? | Two (GC and PP) |
| 25. Description of the coding tree | Did authors provide a description of the coding tree? | Yes |
| 26. Derivation of themes | Were themes identified in advance  or derived from the data? | Derived from the data |
| 27. Software | What software, if applicable, was used to manage the data? | OpenCode |
| 28. Participant checking | Did participants provide feedback on the findings? | No |
| *Reporting* |  |  |
| 29. Quotations presented | Were participant quotations presen­ted to illustrate the themes/fin­d­ings? Was each quotation identified? *e.g. participant number* | Yes |
| 30. Data and findings consistent | Was there consistency between the data presented and the findings? | Yes |
| 31. Clarity of major themes | Were major themes clearly presen­ted in the findings? | Yes |
| 32. Clarity of minor themes | Is there a description of diverse ca­ses or discussion of minor themes? | Yes |
