# Supplementary material for: A qualitative exploration of post-acute stroke participants’ experiences of a multimodal intervention incorporating horseback riding
Source: PLoS One. 2018 Sep 20;13(9):e0203933. doi: 10.1371/journal.pone.0203933 (PMC6147507; doi:10.1371/journal.pone.0203933)
Supplement: S2 Table — (DOCX) [file pone.0203933.s002.docx]

| **Location.** The horse-riding intervention was delivered at a riding center purpose-built for the disabled. Trained therapy horses were used. The sessions were held outside in the paddock or, in bad weather, inside in the ring.  **Staff.** The sessions were led by a physiotherapist and an occupational therapist educated in horseback riding therapy and in stroke rehabilitation. Depending on the level of mobility and capabilities of the participants, there were 2–4 support staff, who helped the participants to mount and dismount from the horse and who also walked beside or led the horses while participants were riding.  **Outline.** Two 240-minute sessions each week for 12 weeks. Each session consisted of riding and time for interaction with the horse either before or after the riding. Lunch or refreshments were served after each session with all staff (leaders and support staff) present.  **Session content.** Throughout the riding lesson, riders engaged in specific exercises individually tailored to their cognitive and physical needs as well as horse-riding ability; all exercises were, if possible, performed while the horse was moving.  **Targeted outcomes.** The horse-riding intervention offered a multisensory environment designed to stimulate and improve motor functions (posture, balance, gait, coordination, muscular and trunk control, body awareness), muscular strength, mental and physical endurance, cognitive functions, attention and concentration, body image, and self-esteem. It also offered enjoyment and social interaction and aimed to provide a sense of mastery. The human–animal interaction also aimed to provide a stress-reducing and calming effect. |
| --- |
