# Supplementary material for: A qualitative exploration of post-acute stroke participants’ experiences of a multimodal intervention incorporating horseback riding
Source: PLoS One. 2018 Sep 20;13(9):e0203933. doi: 10.1371/journal.pone.0203933 (PMC6147507; doi:10.1371/journal.pone.0203933)
Supplement: S3 Table — (DOCX) [file pone.0203933.s003.docx]

| **S4 Table. A summary of themes, sub-themes, codes, and meaning units, relating to the experiences from the multimodal intervention with horseback riding** | | | |
| --- | --- | --- | --- |
| **Theme** | **Sub-theme** | **Example of labelling codes** | **Meaning units** |
| Transformative experience | Beyond expectations | From hamburger to own farm | P1: If I had known this, I would have bought my uncle’s farm. But at that time, it was ‘never’ for me – a big no, no! Horses were hamburgers to me! |
|  |  | Wildest dreams to sit on a horse | P9: I couldn’t have imagined in my wildest dreams that I would ever sit on a horse’s back. It was something that was completely beyond my imagination.! |
|  |  | Lucky that I got ill | P5: Seen from a slightly gloomier perspective, it was lucky that I got ill so I could take part in this [horseback riding], if being ill was the excuse that was necessary. |
|  | Consequences for physical performance | Able to walk longer distances now | P4: So, it [horseback riding] also meant that I am able to walk longer distances now than I did before. |
|  |  | Completely drained of energy | P13: I was so devilishly tired in my head afterwards! Because it was like being in control of myself for just a few minutes… I was completely drained of energy and power after that. That’s why I felt that this was good training for me – this was exactly what my problem was. |
|  |  | Suddenly able to stand in the shower | P7: After six or seven times, I suddenly noticed that I was able to stand up in the bathtub without holding onto anything! Oh! I was so astonished when I stood in the shower, I thought to myself: “Oh my God, I’m not even holding onto some­thing!” |
|  | Impact on self-esteem | Dare to take on bigger challenges if brief | P8: I dare to take on bigger challenges now, as long as I know they are brief. When I know that the challenge is long-standing, I will have to skip it altogether. |
|  |  | It’s about confirming “I can do it!” | P5: It’s very much about confirming that “I can!”. Obviously, this has been a very good experience! [---] It’s really a little extreme, when one thinks about it: sitting on a horse and holding onto it and trotting, and stuff. It gave a kick, and that’s probably important to a lot of people. [---] It gave me an extra reinforcement of my self-esteem. . |
|  |  | I can do more than I thought | P7: I can do more than I thought! [---] When I noticed that it went beyond my expectations, I realised that there might be other things that I could do that I didn’t think I could do. |
| Human–horse interaction | Emotional bonding | Fell in love with the horse | P16: I… fell in love… I think so… (trying to find the words) … no, oh, no [---]. Well, it was very educational that a horse could mean so much to me, I didn’t think so! It’s their eyes. What eyes they have! |
|  |  | Communing with the individual | P8: It was almost as if the horse was, well not a person perhaps, but I wanted to hug the horse (laughs). It felt like communing with an individual! |
|  |  | Grooming leads to close contact | P7: In order to ride, you can’t just sit on top of a horse, you also have to try to get close to the horse and get acquainted with it. When you groom it, you get this close contact and you get this feeling “it’s you and me working together”. [---] That contact is just as important as getting up on the horse.” |
|  | Experiencing the own body through the horse | Following the horses’ rhythm and gait | P9: You learned how to balance. The horse moved and you had to continuously follow the horse’s movements, and that was really useful. You had to follow the rhythm of the horse. You couldn’t just sit there, you had to follow the horse’s gait [---] That’s the whole point of riding bare-back, to gain the balance. |
|  |  | Must first learn about your own body | P1: When you get up there, you must first of all learn about your body as a human being, how it works. Start learning how your body works. |
|  | Learning to master the horse | So much fun to control the horse | P3: I was helped to get up on the horse, I needed help to get the left leg over the withers. But then you could steer the horse by yourself, and that was really fun! |
|  |  | Learning by observing the others | P15: That was also good for me, to see the others develop. That was really posi­tive. Not only for myself, but also to see how the other manage. You sit there and observe and learn. |
|  |  | First times were hard without the control | P13: The first times were a bit hard. I had no real control, I thought, when I was sitting there on the horse… happy as long as I didn’t fall off! |
|  | Enjoying the horse as a co-worker | Doing this together | P4: It’s me and the horse who are doing this together. That felt really nice. |
|  |  | Able to make the horse understand | P5: It was a good thing that I learned how to ride, to work together with a horse. I could make the horse understand my thoughts, and it did what I wanted it to. |
|  |  | Needed to give away the authority | P7: I had to give away the right to decide, and let her control how my body should work, because I had to follow her and not the other way around. |
| Togetherness and belonging | Enjoying the success of the others | Being inspired by the success of others | P3: There was one man, he recovered the ability to grip with his hand during the riding therapy. Those things are really inspiring. |
|  |  | Fantastic sight when his fear let go | P7: X [mentions a persons’ name] who was in my group, he was terrified of horses! He had no previous riding experience, he was so frightened, and he was so good. That day when his fear let go, that was a fantastic sight! |
|  |  | Inspiring to see the others improve | P7: And when I saw her development from having problems walking to being able to walk with ease and being able to trot on her own while on horseback, that was really something! |
|  | Sharing each other’s riding experience | Helped each other to analyse events | P15: We tried to help each other a bit and talked about why things had happened, and why it didn’t go as planned and so on. |
|  |  | Dealing with this afterwards was good | P5: This combination of working with the animals and then having time to deal with the experience afterwards was probably really good! |
|  |  | Gave a sense of landing afterwards | P5: It was so nice and pleasant, because you also had a wonderful feeling of being able to land after the intense session. You were not in any hurry to go home, which also was valuable from a therapy point of view. We were encouraged to take it easy. |
|  | Developing interpersonal skills | Practicing how to socialise  Using the time in a sensible way | P15: And then we had lunch, and we talked about what had happened, but also about other things. Because that’s what happens when you get to know each other better, you start talking about things in general. So, it also became good practice to socialise and to spend the time in a useful way. |
|  |  | If the social doesn’t work the riding won’t | P9: I believe that it’s important that the social part in the little group also works well, it’s not only the riding itself! The part when we ate lunch together was just as important as the riding itself, that’s what I think anyway! |
| The all-in-one solution | The arrangement as such | The all-in-one solution is great fun | P3: Well, the all-in-one solution was great fun when we had lunch together and drank coffee and so on. |
|  |  | Long way to travel by bus is exhausting | P13: The bus travelling was not as nice. I had to transfer to another bus at one place and it took a while. It was very strenuous for me to sit such a long time with people who make a lot of noise and speak on their phones and so on. So, it… very tiring. |
|  |  | Energy demanding to be early (trans. serv.) | P14: I was sometimes a bit early and then it was… hard to sit on that chair, you know [in need of travel service]. |
|  | Competent and devoted instructors | Professional leaders who adapted tasks | P9: They had to adapt a bit for this group, I think so. And they are so professional, they can manage this. |
|  |  | Knowledgeable and engaged instructors | P4: They were very knowledgeable, engaged persons. So, it was a very positive surprise. The experience was very good, very light. |
|  |  | Leaders were familiar with stroke | P13: It was obvious that they knew what sort of people they were dealing with, they were very familiar with the problems one might have. That it can be so different from person to person. |
|  | In comparison to other training forms | Self-training is often fragmented | P5: Exercising on your own becomes very fragmented and difficult. But with horseback riding therapy you do it! You don’t have to think, you are in a different place with other people who do the same thing, and you just focus on the training itself. If you work out at home, it gets fragmented. |
|  |  | Bicycling/jogging pointless, but horse great | P6: And bicycling – completely useless! I used to be a professional bicyclist, you know, been cycling about 200 km a week or more. And I used to run 30 km a week. That sort of thing doesn’t do anything for me – but the horse, so damn good! |
|  |  | Important to combine with arm-training | P5: The horseback riding isn’t directed towards any particular body part … I mean like working out with a shoulder or an arm – I think that has to be done as well. |
