# Supplementary material for: A qualitative exploration of post-acute stroke participants’ experiences of a multimodal intervention incorporating horseback riding
Source: PLoS One. 2018 Sep 20;13(9):e0203933. doi: 10.1371/journal.pone.0203933 (PMC6147507; doi:10.1371/journal.pone.0203933)
Supplement: S1 Appendix — (DOCX) [file pone.0203933.s004.docx]

# Background and study context

Qualitative interviews were conducted in the context of a randomised controlled trial (RCT) entitled ‘Long-term improvements after multimodal rehabilitation in late phase after stroke’.

## decisions taken prior to the present study

The philosophical position underlying this study relied on an interpretivist orientation to qualitative inquiry, as opposed to the positivist orientation driving the RCT. The ontological position of interpretivism is relativism, which is the view that reality is subjective and differs from person to person (Guba & Lincoln, 1994). Based on the research question, an interactionist perspective was adopted as the methodological approach where people are seen as actively participating in the creation of their own development through interaction with the social world. This relational approach deals with how people shape their realities through in­ter­action and interplay with other people in relation to a social context (Mead, 1962; Blumer 1986).

The interpretivist approach is based on a naturalistic approach to data collection, and therefore, as data source, it was decided to use individual face-to-face interviews. To guide the interpretation of the data, a qualitative content analysis was chosen as method of analysis (Graneheim & Lundman, 2004). Given the study aims, we chose an inductive approach when analysing the data. With an inductive approach, little or no predetermined theory, structure, or framework is used to analyse data. This approach is recommended when there is little former knowledge about the phenomenon of interest. Thus, the themes were derived from the data and not on the basis of previous knowledge or theories.

These philosophical and theoretical standpoints form the basis for how we chose to view and understand the social worlds and interactions within the intervention groups.

### Planning the interviews

When constructing the interview schedule, the research team discussed possible main questions and prompts that could be used in order to identify participants’ perceptions and experiences from taking part in the group-based active interventions. Emphasis was placed on understanding the individuals and their interpretations of their experiences from the intervention. It was also emphasised that the questions should be neutral rather than value-laden or leading. The first draft of the interview questions was considered to be too explicit and linked to specific assumptions. The second draft of the semi-structured interview schedule was then constructed, with the follo­w­ing questions:

- Describe in your own words your experience of participating in the horseback riding group. Positive or negative experiences?
- Has this participation had any effect on your physical, psychological or social abilities?
- Has participation meant something for you in your contact with other people? In connection with horseback riding group? In other contexts?
- Has participation affected your activity performance in life in general?
- Has participation affected your life situation in general?
- Has participation affected your mood, quality of life, and beliefs about the future?

Prompts were given to encourage participants to expand upon their feelings and thoughts, such as “In what way was it so?”; or “Could you elaborate how you mean more specifically?”. One pilot interview was conducted, which was later included.

### Sample size

A pragmatic approach to the determination of sample size was used instead of using data saturation because of the rather narrow research question, which was to explore participants’ experiences of the horseback riding. It was esti­ma­ted *a priori* that between 15 and 20 participants would be suffi­ci­ent to capture a variety of expe­ri­en­ces.

### Participant recruitment

During the first physical follow-up assessments, all 41 participants were asked if they would agree to be interviewed face-to-face within four weeks after the intervention and in groups after six months, with the motivation that physical assessments and ques­tionnaires would not suffi­ci­ently capture their personal experiences and perceptions of taking part in the multimodal inter­ven­tions. All partici­pants from the horseback riding group agreed to be contacted, except three: one woman refused because she had only been horse­back riding a few times due to back problems; one man planned to take vaca­tion abroad for up to half a year; and in one case a wife refused on behalf of her husband, because she believed he would not be comfortable about being interviewed due to his severe aphasia.

# audit trail for the present qualitative study

The following audit trail solely concerns the research procedure for the individual interviews con­duc­ted with participants from the horseback riding intervention groups. The research question for the present study was to explore stroke survivors’ personal experiences of taking part in a multimodal group-based horseback riding intervention. The study is therefore based on the face-to-face inter­views within four weeks after the intervention.

## Research team and reflexivity

Five researchers were involved in the study. The researchers have different professional back­grounds and expertise from different fields of health care. This was expected to broaden the inter­pretation of the analysis. PP is a physiotherapist and PhD; GC is an occupational therapist and PhD; LKB is a physio­therapist and PhD; MN is a physi­cian and university professor; and CB is a physician and university pro­fes­sor. All re­sear­chers have previous experience from stroke rehabilitation in different settings inclu­ding different cities and countries.

Two researchers (GC, PP) have formal training and several years of experience in qualitative me­thods; the other authors are experienced but have no formal training in qualitative methods. None of the researchers have practical experiences from horseback riding therapy, but there was a pre-understanding within the research team that multimodal rehabilitation may be beneficial in a late phase of recovery. This might have influenced the interpretation and analysis of the data.

All team members read the interviews, but GC and PP conducted the preliminary analysis of the collected data. Consensus was reached during several team meetings.

No relationship was established between the interviewer GC and the participants prior to this study. A speech therapist who was employed to support some interviews had met two of the participants with aphasia prior to the interviews. She did, however, not participate in the analysis of data. No other personal biases were identified before the interviews. The setting for the interviews was chosen to ensure confidentiality in a relaxed atmo­sphere.

## Sample selection and participant recruitment

Purposive sampling was undertaken by GC, and 18 participants out of the 38 who had agreed to be contacted were selected. The sample included parti­cipants from all eight horseback riding groups, males and females with varying ages, levels of dis­ability, with and without aphasia, and with various perceptions of improvement from the intervention. Participants were contacted by telephone by GC. There were no monetary incentives.

## Data collection

Interviews were conducted between December, 2010 and August, 2013 at a rehabilitation facility at Sahlgrenska University Hospital in Gothenburg, Sweden. One pilot interview was conducted, and no adjustments were made in the question guide. The pilot interview was included as one of the inter­views. Seven­teen further participants underwent one face-to-face semi-structured interview with GC, or a speech therapist, or both together. The speech the­ra­pist was there to support the inter­pretation of the participants with aphasia, and conducted two of the interviews on her own. The interviews lasted 17–50 minutes, all interviews were audio recorded using a portable digital audio recorder, and one interview was also visually recorded in order to facilitate the interpretation of the participant’s utteran­ces.

## Data transcription

The interviews were continuously transcribed verbatim in Swedish between February, 2011 and July, 2014 by a person external to the research group and without influ­ence over the research process. One interview was tran­scribed by the speech therapist. Utterances such as ‘mm’, ‘eee’, emphasised words, pauses, or laugh­ing were included.

The transcripts were verified for accuracy by PP (who is also a trained medical secretary), by cross-checking the tran­s­­c­ripts with audio­tapes. The texts were somewhat shortened in that utterances such as the above were removed from the transcribed texts if they were made by the interviewer. No member checks were con­ducted for comment or correction. All transcripts were anonymised by a unique number prior to the analysis (P1 to P18).

## data analysis

Following the previous decision, a qualitative content analysis was used as method of analysis as descri­bed by Graneheim and Lundman (2004 and 2017). This method has been recog­nised as appro­pr­iate when the aim is to focus on the sub­jects as well as on the contextual meanings. The emphasis lies on describing variations, e.g. simi­la­ri­ties and dif­fe­ren­ces within parts of the text. Qualitative con­tent analysis may comprise both descriptions of the manifest contents (close to the text), as well as of the latent messages (close to the lived experien­ces, but more distant from the text). In this study we have mainly described the manifest contents, which, however, may also require some degree of interpreta­tion. The 18 transcribed interviews were considered as the unit of analysis.

The process of coding the material and deriving themes was done in a step-wise process between March and September, 2017.

**Step 1**. All interviews were initially read repeatedly and independently by PP and GC to start the pro­cess of identifying meaning units and to acquire a good grasp of the whole. Meaning units are com­prised of a few words or sentences that correspond only to the research question, i.e., how the horseback riding intervention was perceived and experienced. At times people would talk about things that were not considered to be relevant for the study purpose, such as a pleasant journey. Those sections were ignored.

**Step 2.** Next, the same two authors compared their meaning units, and when differences appeared, they were discussed until agreement was reached. During the process of comparing, asso­ciations were made, and ideas and thoughts were noted in the margin of the texts.

**Step 3.** All text units (i.e., raw data) were formatted into txt. -files and transferred to a computer software system (Open Code 4.0, freely available from Umea University at <http://www.phmed.umu.se/enheter/epidemiologi/forskning/open-code>) to be further processed.

**Step 4.** All meaning units were condensed into labelling codes by both authors together (example in Table 1). A code is close to the text (manifest level) and describes the content of the meaning unit with a few words without losing the core content of the unit. Memos were put into the computer system when initial ideas of themes developed.

| Table 1. Examples of meaning units and the labelling codes. The meaning units and codes were conducted in Swedish, but have been translated into English. | | |
| --- | --- | --- |
| **Code** | **Meaning unit** | **Labelling code(s)** |
| P1 | *“It was perfect, you know, more or less all body parts were active because you sat on… you didn’t have a saddle but instead more or less bare-back riding.”* | - All body parts were involved  - Close contact with the horse |
| P5 | *“In my opinion this has been a positive experience, regardless of my state of health. I mean, seen from a slightly gloomier perspective, it was lucky that I got ill so I could take part in this [horseback riding], if being ill was the excuse that was necessary. This has been great, so I have severe abstinence now that it’s all over.”* | - Positive whether or not ill  - Severe abstinence afterwards  - Lucky that I got ill! |
| P11 | *“My wife has a horse, she would take care of it all by herself! She could never interest me in going to the stables with her before. But now!”* | - Interest in horses increased |
| P16 | *“But afterwards, when we had removed the helmets… oh… and got to feel the horse and caress him. Oh, one could… oh…. Goodness, it felt so good (with emphasis)!”* | - Wonderful to caress the horse |
| P17 | *“We didn’t have any saddles, I think that was exciting. I’m happy that I accepted this activity now. Earlier I said to myself – I will never ride again, I couldn’t care less. But, well… there were so many nice and fun people as well. And we rode for about 30 minutes. And they were so clever, the instructors!”* | - Exciting without saddles  - Had decided never again  - Nice and fun people  - Clever instructors |
|  |  |  |

**Step 5.** The codes were printed on paper and cut into pieces (appr. 500 codes). The codes were there­­­after grouped according to underlying patterns that indicated similarity in contents and labelled with a primary headline describing their con­tents (Figure 1). This was done on a whiteboard with the advantage of freely moving the codes. The process of grouping codes was conducted through several meetings between GC and PP, while continuously going back to the original texts.


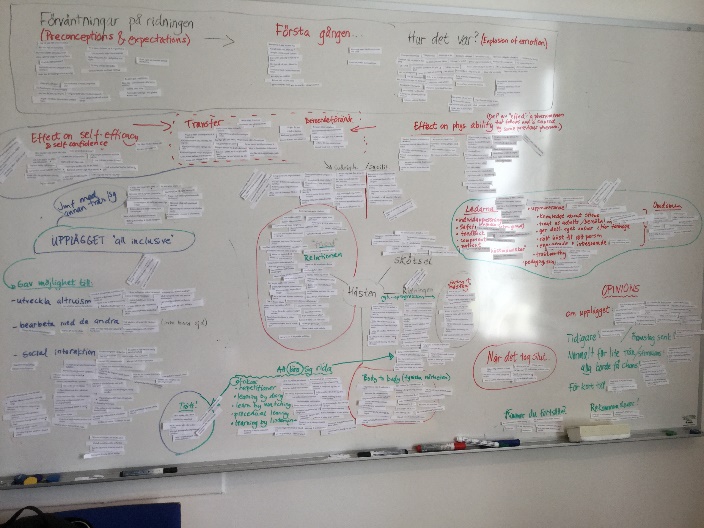


Figure 1**.** Example of how codes were moved around on a whiteboard in order to find underlying patterns

In May, 2017, initial themes started to take form:

**Key factors for success**:
- social integration (the other group members; lunch; pep talking; enjoyed the progress of others)
- instructors (natural concern/empathy; planning the sessions; competent; choice of horse; security, knowledgeable about stroke and horseback riding; treated us as equal adults)
- time in stable (grooming; partnering with peer; petting the horse and making close contact)
- horseback riding (challenging tasks; individually tailored; sense of mastery)

**The design of the sessions**:
- “all-inclusive”-solution: great with horseback riding AND lunch together
- boring to be the last one to ride – long wait vs. time well-spent on socialising

**A strong desire to continue**:
- expensive
- lack of skilled assistance/escort
- missing this very much – “abstinence”

In July, 2017 the themes had evolved:

**Human-human interaction / interpersonal perspectives**
- sharing each other’s experiences by observing
- enjoying the progress of the others
- developing interpersonal skills (socialising during lunch, coffee,)
- waiting for their turn (talking, getting to know each other in a small group, encouraging)

**Human-horse interaction**
- bonding with the horse (time in stable and riding)
- learning how to ride (not just sitting on a horse)
- enjoying the horse as a co-worker – the collaboration
- great to ride without the saddle 🡪 close contact

**Reflections on the arrangement as such**- all-inclusive solution was great (coffee, lunch, riding etc)
- everybody should be given this chance
- too short
- expensive to continue
- comparisons to other rehabilitation activities
- great instructors

**Transformative experiences**- beyond wildest imagination (sad when it ended)
- improved balance and other physical body functions including body awareness
- increased self-efficacy (I can do this!; encouraged by the others and by the instructors)

**Step 6.** In August, 2017, a first set of themes and sub-themes were presented along with the codes to the other three team members (LBK, MN and CB) (Table 2 and 3). Their role was to consider the ana­lysis in relationship to the transcribed data and to comment on the labelling of themes as well as the appro­priateness of the themes and sub-themes, respectively. Notes were continuously taken during the meeting. A first preliminary sketch was also developed during this meeting where the horse was central, which was later created in a computer programme (Figure 2 and 3).

| Table 2. First suggestion for themes, sub-themes and brief contents (August, 2017) – short form presented to the other authors. | | |
| --- | --- | --- |
| **Themes** | **Sub-themes** | **Brief contents** |
| Transformative experiences (changing the view on  oneself and the world) | Beyond expectations | Preconceptions |
|  |  | First time |
|  |  | Explosion of emotions |
|  |  | After the intervention |
|  | Physical performance as a consequence | Impact on hand function |
|  |  | Impact on balance and strength |
|  | Impact on self-esteem | Improved self-esteem |
|  |  | Fear and shame |
|  | Courage to make changes | Developed courage |
|  |  | Actual behavioural changes |
| Human-horse interaction | Emotional bonding with the horse as a friend | In love with the horse |
|  |  | Mutual understanding |
|  | Experience the body through horse’s | Physical closeness to the horse |
|  | Learning how to ride and  master the horse | Learning by observing |
|  |  | Learning by doing |
|  |  | Learning by listening |
|  |  | Focus / concentration |
|  |  | Progression |
|  |  | Feeling of mastery |
|  | Enjoying the horse as a co-worker | Working together |
| Interpersonal perspectives | Sharing each other’s riding experience | Processing the experience together |
|  | Enjoying the success of the others | Observing each other’s proceedings |
|  | Developing interpersonal skills | Social interaction |
|  |  | Fun together |
|  |  | Co-work in the stable |
| The all-inclusive solution - reflections and opinions | The arrangement as such | About the arrangement |
|  |  | Energy demanding |
|  | Competent and dedicated/devoted instructors | Choice or horses; tailoring; engaged |
|  | In relation to other training forms | Compared to physiotherapy |
|  |  | Organized vs. home-training |
|  |  | Early vs. late |

| Table 3. First suggestion for themes, sub-themes and detailed contents of all labelling codes (August, 2017) – long form presented to the other authors. The labelling codes have been kept in Swedish in their raw form to enhance transparency. | | |
| --- | --- | --- |
| **Main themes** | **Sub-themes** | **Labelling codes and short description** |
| Transformative experiences (förändra sin syn på sig själv och världen) | Beyond expectation (över förväntan) | Preconceptions: Från hamburgare till egen häst­gård, hade jag varit frisk hade jag köpt en häst!; hästar är hamburgare; Inte i vildaste fantasin kunna tänka sig att rida; Ni är galna i huvudet – jag är gammal; åka häst kan jag väl göra; hästar ingår inte i mitt liv normalt; tidigare tyckte häs­tar luktar illa (havremoped); negativ till hästar innan terapin (förbjöd barnen); tyckte hästar luktade och kostade pengar; Hade inga förvänt­ningar; Glad att något händer |
|  |  | Första gången: kastade mig in med huvudet före; svindlande känsla innan – hästen är stor; herregud – komma upp på den klumpen; blev lugn när väl uppe – hästen fin; kände mig osäker från början men inte rädd; respekt innan |
|  |  | “Explosion of emotions”: Gav mycket värme och positivitet; längtade till nästa gång; en av de bätt­re grejerna som hänt mig; Jätte­glad att jag ändrade mig; jättenyttigt för mig; upplevelsen var väldigt bra, väldigt ljus. Tur man blev sjuk så man fick möjlighet att vara med! |
|  |  | Efter interventionen: väldigt väldigt ledsen när vi slutade; blev ledsen när det slutade; synd att inte hålla det levande; svår abstinens; tappade lusten till aktivitet efter interventionen; |
|  | Physical performance as a consequence | Balans och styrka förbättrades: blivit säkrare och bättre balans; hållningen har blivit bättre; förbättrad styrsel (flaggstång innan); kunde gå i trappa utan stöd; orkar gå längre sträckor; kas­ta­de käppen; **märkte ingen skillnad i höger sida** |
|  |  | Bättre finmotorik: Sköta hästen och tömmar förbättrade finmotoriken; fick mer motivation att rida för handen blev bättre; |
|  | Impact on self-esteem (påverkan på självförtroendet) | Ökat självförtroende: Den stora grejen var bätt­re självförtroende; en kick av känslan ”jag kan fast jag är sjuk!”; Ändrar ”jag kan inte” till ”jag kan”; jag blev mallig; handlar om att bekräfta ”jag kan”; jag lyckades komma så långt; omgiv­ningen märkte stor skillnad; jag vågar lita mer på mig själv; mer säker på sin egen förmåga – ställer krav; rädd att trava och göra balans­öv­ning; skäms att hon ramlat av som ridit så mycket; jämför med sig själv innan (var duktig ryttare innan stroken) |
|  | Courage to make a change (fått mod att förändra saker) | Vågar förändra: vågar ta större utmaningar; fått bättre mod att säga ifrån; vågar göra mer saker; springer och visar barnen på fotbollsplan; rid­ning­en öppnade för nya möjligheter; vågade börja snickra efter ridningen; jag vågar göra mer saker; vill gärna testa nya saker; kan jag rida kanske jag också kan göra andra saker; har fort­satt att rida – men saknar det sociala; vågar se framåt – kommer träna sitt barnbarn; vill gärna fortsätta rida; återskapat intressen och vågar prova nya intressen |
|  |  | Beteendeförändringar: Kickstart till att börja banta; börjat träna balansen i gymnastiken efteråt; förbättrad balans spillt över på andra aktiviteter (segling); börjat rida på sin frus häst – ridningen blev gemensamt intresse. Blev mer aktiv; Hästar har blivit ett intresse; |
|  |  |  |
| Human-horse interaction | Emotional bonding with the  horse as a friend | Jag tror jag har blivit kär i hästen!; Att en häst kan betyda så mycket!; bekräfta hästen; kom­mu­nikationen med hästen var stort; kände stark gemenskap med hästen; gemen­skapen med häs­­ten var största behållningen; det är du och jag hästen!; skötsel ger närkontakt; trevlig del att sköta om hästarna; givande samvaro med hästen; fick så mycket tillbaka; extra kul med djur – hästar är väldigt påtagliga; respekt för hästar – kom väl överens; man gullar inte med hästar i knäet; hästarna upplevdes som snälla; blev kompis med hästarna; min häst förstod mig precis |
|  | Experience the own body  through the horse’s | Fysiska närheten till hästen: lära känna sin kropp på hästryggen, följde hästens rytm och hästens gång; rida utan sadel för när­kontakt och balans; låta hästen styra hur min kropp skall fungera; hade varit enklare med sadel; kom in mer och mer i hur hästen rörde sig; lärde mig mer om att hantera kroppen; alla kroppsdelar är med; hela kroppen fick en kick; känner sin kropp bättre |
|  | Learning to ride and  mastering the horse (att lära sig rida) | Genom att observera de andra: lärde mig genom att observera de andra; positivt inte bara för mig – ser hur andra gör; lärde sig av att titta på de andra; tittar och observerar de andra; roligt att titta på de andra |
|  |  | Learning by doing: har lärt mig att rida inte bara åka häst; som att lära sig cykla; automatisering gjorde att hon kunde hitta på egna vägar; Repetitioner: innötning förbättrade minnet; träningen skedde med innötning; |
|  |  | Genom att få instruktioner: lätt att lära sig bara man lyssnar |
|  |  | Fokus: kräver total närvaro; kräver koncentra­tion; tappar man koncentrationen blir det fel; minnesproblem blev en begränsning; blev fel en massa gånger – glömde; svårast att memorera en bana |
|  |  | Progressionen: i början fick man klamra sig fast/hålla sig kvar; i början fantastiskt att klara av att sitta kvar på hästen; ingen dramatisk utmaning i början – försiktigt; gick bättre och bättre; fick byta till häst med lite mer liv i; man utvecklades och höjde ribban; |
|  |  | Feeling of mastery: skoj att rida själv; skitskoj att styra hästen själv; roligt få vara den som be­stämmer; väldigt skönt att själv ha kontroll; ro­l­igt få göra hästen att göra som jag ville; gick bra att styra hästen själv; en kick av att trava och hål­la sig kvar; bra att hästen gjorde som jag ville; roligare att få rida själv inne än att få hjälp ute |
|  | Enjoying the horse as a co-worker | Bra att jag kunde jobba tillsammans med häs­ten; samarbete mellan mig och hästen (trygg); bra att jag kunde få hästen att förstå hur jag tänkte; det var så kul att börja jobba med häs­ten; interaktionen med hästar gav mycket; häs­ten och jag gör detta tillsammans; svårast att kliva upp på hästen; svårt komma ner pga. övervikt**;** var tvungen att lita hästen; hästarna gör jobbet; att ge bort bestämmanderätten; |
|  |  |  |
| Interpersonal perspectives | Sharing each other’s riding experiences | Bearbeta med de andra: viktigt att få prata efter ridningen; positivt att snacka efteråt; jobba med djur och hantera efteråt är bra; försökte hjälpa varann att analysera ridningen; fick känsla av att landa efter ridningen (lunch) |
|  | Enjoying the success of the others (peers enhance the learning experience with positive reinforcement) | Observerar andras framgångar: Fantastisk syn när hans rädsla försvann; roligt att se hur andra avancerar; njuter av att se andras utveckling; bekräftar förbättringar hos varandra; gav alltid de andra ryttarna beröm; att se förändringen hos andra var enormt; sporras av andras fram­gångar; viktigt att höra om andras framgångar; medvetet sporra andra med positiva kommen­tarer; noterade hur andra red och gav feedback efteråt; förvånad över andras framgångar, att från ingenting komma så långt (Bill) |
|  | Developing interpersonal skills (väntetid på bänken, lunch, hästskötsel) | Social interaktion: En övning att lära sig umgås – ta vara på tiden; samtalen med de andra gav väldigt mycket; jättebra sitta och prata trots tal­svårigheter; väldigt skönt att få tala lite grand (afasi); hade det hur bra som helst (mat och läktare); viktigt att det fungerar socialt mellan deltagarna; värdefullt göra aktiviteten till­sam­mans med andra; olika sociala klasser – katt bland hermelinerna; sociala biten var fantas­tiskt; viktigare med det sociala än med rid­ning­en; vi lärde känna varann – saknar det; blev sam­mansvetsade kände ej varann från början; positivt att träffa andra som råkat ut för samma sak; kan visa sina svagheter för de andra; funge­rar inte det sociala fungerar inte ridningen; om någon tog över samtalet pratade man med någon annan; skönt att slippa prata om stroke; pratade om allt annat än sjukdomar; kände mig inte bortkommen eller konstig; rida, inte grupp­en var drivkraften till att åka dit; ingen bestå­ende grupp – bara ridningen; bara två i grupp­en – ej givande socialt |
|  |  | Roligt ihop: vi skrattade gott; jättebra göra kul grejer med andra; träffade några olycksbröder och hade roligt; |
|  |  | Hästskötsel: samarbete kring hästskötsel; minst lika roligt med skötseln av hästen; deltagande + närstående hjälptes åt; Samarbete kring hästskötsel (Bill o jag resp. en tjej och jag); |
|  |  |  |
| The all-inclusive inclusion  – reflections and opinions | The arrangement as such | Om upplägget: Upplägget var bra; högsta betyg för hela arran­gemanget; var rädda om upplägg­et; viktigt med rutiner; helhets­lösningen var him­la kul; hela upp­lägget och arrangemanget var bra; hel­hets­lösningen gav mycket; både so­ciala spelat och det praktiska var viktigt; upp­lägget är tryggt och säkert; jättetrevligt – mat och människor; det får de bara inte sluta med!; bra att göra en sak ordentligt; hoppas fler får chansen!; inte bara för strokepatienter utan ger mycket mer; ridning stor hjälp för de som har intresse och vilja; kan göra mycket fast man är sjuk; tråkigt att rida sist; roligt att rida men tråkigt att vänta på sin tur |
|  |  | Energi- och tidskrävande: blev mycket trött efter­åt; trött i huvudet efteråt; så in i bängen trött efter en liten stund; blev helt dränerad efter några minuter; trött i början men gick över; mindre behov av att vila ofta; möjligen mindre trött i huvudet; stress pga. tidskrävande; lång dag väldigt trött vid hemkomsten; resorna innebar för mycket stimuli |
|  |  | Utmanande övningar: Bra med ökande pro­gres­sion och ansvar; lades på uppgifter successivt; nyttigt att träna det man har problem med; vik­tigt att få utmanande övningar; sporras av ut­ma­ningar; kul med utmaningar; strokedrabbade kan mer än vad folk förväntar sig; hade varit roligare att få rida fritt; tråkigt att göra samma saker; blev lite inrutat med samma mönster; hade velat jobba mer med ridningen; blev inrutat först – trodde mer fri ridning |
|  | Compared to other training forms | Jämfört med sjukgymnastik/rehab: detta kan inte en sjukgymnast träna; en helt annan grej än sjukgymnastik; mer komplext än vanlig sjuk­gym­nastik; lite tuffare än annan rehabilitering; bästa rehabiliteringsgrejen; som terapi har ridningen betytt mest; annan rehabilitering försiktighets­moment – det här är lite mer; rid­ning borde ingå i reha­bilitering; många vill göra mer än de får tillfälle till; |
|  |  | Organised training vs. home-training: viktigt med organiserad terapi vs. hemträning; lättare att träna med organiserad aktivitet; självträning blir splittrad och svår efter stroke; kvaliteten blir bristande av självträning hemma |
|  |  | Early vs. late initiation: Viktigt starta med en gång; viktigt att rida tidigt i rehabiliteringen; man kan faktiskt göra fram­steg många år efter; man är aldrig för gammal att lära nytt; bekläm­mande när behövande bara får tidning; |
|  | Competent and dedicated/devoted instructors | Skräddarsydde träningen för varje individ; duk­tiga ledare; rätt häst till rätt person; kunniga och intresserade ledare; peda­gogiska ledare; bra med instruktör som säger ”gör det”; positiv känsla att man visste vad man skulle göra; le­dar­na tillrätta­visade bra; ledarna var också häst­människor; kunniga och engagerade; le­dar­na mötte oss som vuxna; ledarna väl in­satta i pro­blemen; viktigt med visst allvar – men inte blo­digt (lek, trevligt); ledarna var delaktiga hela tiden; lugnt och trevligt – ledarna hade kontroll; litade till fullo på ledarna; alla fick en chans att lära sig oavsett förmåga; fick jobba efter egen förmåga; individ­anpassad träning; alla utveck­lades oavsett funktion; det var olika men bättre för alla; underbara/fina/trevliga; sån personal måste ni vara rädd om; ledarna var noga med säkerhet; tålmodiga; kände oss jämlika med ledarna – mötte oss som vuxna |


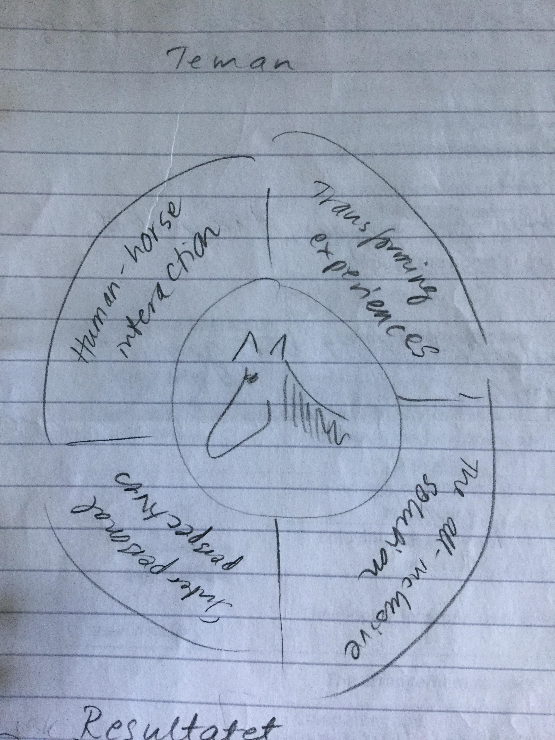

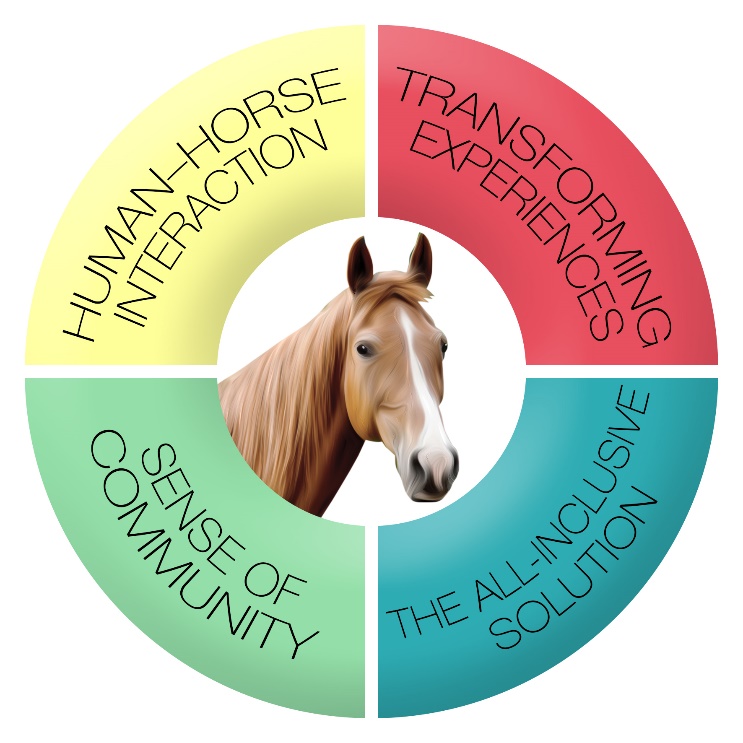


Figure 2 and 3. First draft of the model

Following this discussion, further analysis was conducted by GC and PP, while re-reading the transcriptions.

**Step 7**. A second set of themes and sub-themes were presented to the research team. The final set of themes was determined by consensus. The model was revised accordingly.

**Step 8**. Manuscript was written by PP and GC and edited by LKB, MN and CB, reporting followed the recommendations of COREQ (Tong et al)
